# Proportion of Hidden Vertebral Fractures Among Egyptian Males With Fragility Hip Fractures in the Emergency Room of Ain Shams University Hospitals

**DOI:** 10.7759/cureus.49960

**Published:** 2023-12-05

**Authors:** Adel A Abbas, Mohamed F Allam, Hala S Sweed, Samia A Abdul-Rahman, Walaa W Ali

**Affiliations:** 1 Geriatrics, Ain Shams University, Cairo, EGY; 2 Preventive Medicine and Public Health, University of Cordoba, Cordoba, ESP; 3 Family Medicine, Ain Shams University, Cairo, EGY

**Keywords:** ain shams university, egypt, male, vertebral fracture, osteoporosis, hip fracture, fragility fracture

## Abstract

Background

Fragility fractures are linked to significant costs for society as well as significant pain and suffering, disability, and even death. It is well-recognized that osteoporosis-related fragility fractures raise the risk of subsequent fragility fractures. One of the most frequent osteoporotic fractures brought on by low bone mineral density and other risk factors is vertebral fractures. Considering that most vertebral fractures are asymptomatic and not clinically identified, proactive screening could stop additional impairment.

Objective

The current study aims to determine the prevalence and risk factors of hidden vertebral fractures in Egyptian males who have fragility hip fractures.

Patients and methods

A cross-sectional case-control study examining the correlation of risk factors between cases (fragility hip fracture and vertebral fractures) and a control group (fragility hip fracture without vertebral fracture) was carried out from September 2020 to September 2021 on patients visiting the orthopedic emergency department of a university hospital in Cairo, Egypt. Males who presented to the emergency room (ER) with fragility hip fractures and were 40 years of age or older met our inclusion criteria. For every patient who presented with a fragility hip fracture, standard lateral and anteroposterior radiographs of the dorso-lumbar spine were taken.

Results

A total of 43,935 patients visited the orthopedic emergency room (ER) throughout the study period; 13,034 of those patients were men, accounting for 29.7% of all orthopedic ER visits. Our inclusion criteria for fragility hip fractures were met by 132 male participants. The screening lumbosacral plain X-rays identified 27 (20.5%) of the 132 patients as having concomitant vertebral fractures in addition to the fragility hip fractures. Concomitant hidden vertebral fractures among Egyptian males with other fragility fractures, particularly fragility hip fractures, are predicted by the number of co-morbid diseases, hypertension, and continuous use of steroids and anti-epileptics.

Conclusion

Most fragility fractures are avoidable. Because one fragility fracture increases the likelihood of others, early detection is crucial. To prevent complications and mortality, it is important to identify and manage individuals who have a fragility hip fracture as they frequently have concurrent hidden vertebral fractures. Predictive risk factors for fragility vertebral fractures include hypertension, the number of concomitant illnesses, and chronic drugs (anti-epileptics and steroids).

## Introduction

It is well-recognized that osteoporosis is a serious health issue, affecting 200 million people globally [1]. Osteoporotic (fragility) fractures are expected to occur in about 40% of females and 20% of males with osteoporosis during their lifetime [2]. Osteoporotic fracture-related mortality is between 15% and 30%, which is comparable to the rates of stroke and breast cancer [3].

With life expectancy continuing to rise, the prevalence of osteoporosis is rising dramatically and becoming a significant public health issue; this is especially true in poorer nations [4]. By 2050, there will be more than 130 million people living in Egypt, and more than 30% will be 50 years of age or older [5]. The prevalence of osteoporosis in Egypt was 21.9% in men and 28.4% in women in 2018 [6].

Osteoporosis incidence varies greatly between countries based on factors such as population age, sex, and ethnic distribution, and the lack of information regarding epidemiology in the Middle East makes estimation of its incidence more challenging [7].

Age is a major contributing factor to the prevalence of vertebral fractures, which affects 7.5% of men under 60 and 20% of men over 70 [8]. The frequency of vertebral fractures may be underestimated because many of them are asymptomatic, and people may not seek medical attention for them. Serious morbidity, such as back pain, kyphosis, and height loss, are linked to vertebral fractures and significantly lower quality of life, as determined by quality-of-life scores [9].

Studies showed that people with at least one vertebral body fracture had a higher mortality risk than people without such fractures [10]. Rather than being directly injured from falling, most vertebral fractures in the elderly are caused by everyday tasks such as lifting and bending over [11].

The current study aims to determine the prevalence and risk factors of concurrent hidden vertebral fractures in male Egyptian patients who visited the orthopedic emergency department (ER) of Ain Shams University Hospitals (ASUHs) with fragility hip fractures.

## Materials and methods

This cross-sectional case-control study included patients who visited the orthopedic emergency rooms of Ain Shams University Hospitals (ASUHs) in Cairo, Egypt, over a one-year period, from September 2020 to September 2021.

This study's inclusion criterion was patients (males 40 years of age and older), presenting with fragility hip fractures; individuals who did not meet the previously stated inclusion criterion were excluded.

Consecutive sampling was the sampling strategy used. We conducted a thorough investigation on a simple random sample of males who met our inclusion criterion for fragility hip fracture.

Based on data from ASUH and the number of patients registered for ER visits over the preceding five years, an estimated sample size was determined. Ninety-five percent was the confidence level and 3% was the precision level. About 2.3% of male patients with hip fractures were 40 years of age or older. There were 96 patients in the preliminary sample. There were 15% of projected lost follow-up patients or patients with missing data, and 112 patients made up the final sample size (approximated to 115). According to the following formula: Sample Size n = N * [Z^2^ * p * (1-p)/e^2^] / [N - 1 + (Z^2^ * p * (1-p)/e^2^], where* *N = Population size, Z = Critical value of the normal distribution at the required confidence level, p = Sample proportion, and e = Margin of error. A total of 132 patients who met the inclusion and exclusion criteria were enrolled during the research period.

Every participant underwent a thorough evaluation. We collected all male patients presenting with fragility hip fractures aged 40 or older during the appointed period. Hip fracture was diagnosed using X-ray imaging criteria as interpreted by radiologists in ASUHs. Fragility hip fracture is identified as a fracture resulting from a fall from standing height or less. A detailed history was taken including all medical and sociodemographic characteristics (age, marital status, education level) and cause of presentation to the emergency department, including hip fractures, whether fragility fracture or not, to omit participants with high impact fractures or other pathological fractures. Risk variables for fragility hip fractures were evaluated and included age, estimated weight in kilograms, calculated length, rheumatoid arthritis, and secondary osteoporosis. Drug history, especially for steroids (≥5 mg/day prednisone or equivalent for ≥3 months) and current anti-epileptic use for at least six months, was taken.

Our definition for educational level in this study is classified as: low education (having completed fewer than six years of formal education), mid-education (having completed six years of education or more but not a university degree), and high education (having completed a university degree or its equivalent).

An approximation formula based on body height, waist circumference, and hip circumference was used to estimate body weight. In times when the participant’s exact weight could not be measured quickly, we used this approximation method instead of visual estimation [for men, height was measured in cm - 100 - (height in cm - 150)/4] [12]. Using a ruler, height was measured while standing straight up and without footwear. The measurement was taken to the closest half-centimeter. When an older person's inability to stand steadily or straight due to a fracture, discomfort, weakness, or spinal abnormalities, made it difficult to measure their standing height, this formula [Height = 57.345 + 2.131 (Knee height in cm)] was used to estimate standing height [13].

Body mass index (BMI) was then calculated and classified: underweight (BMI < 18.5 kg/m^2^), normal (18.5 kg/m^2^ ≤ BMI < 25 kg/m^2^), overweight (25 kg/m^2^ ≤ BMI < 30 kg/m^2^), and obese (BMI ≥ 30 kg/m^2^) according to World Health Organization criteria.

Conventional lateral and anteroposterior X-ray radiographs of the dorso-lumbar spine were done as determined and diagnosed by a radiology specialist utilizing the semi-quantitative approach, for patients presenting with acute fragility hip fractures to screen for concurrent (old or recent) hidden vertebral fracture [14]. In this method, vertebral fractures are graded from 1 (mild) to 3 (severe). A reduction of about 20-25% in anterior, middle, and/or posterior height relative to the expected normal vertebral height indicates a grade 1 (mild) vertebral fracture. Vertebral height reductions of approximately 25-40% are considered grade 2 (moderate) fractures, whereas reductions of approximately 40% or more are considered grade 3 (severe) fractures.

Consumption of alcohol was measured by direct question and was categorized as either alcoholic (regular intake of alcohol, three or more units/day) or non-alcoholic. Self-reported smoking status was summarized into nonsmoker or current smoker (daily or occasional). Patients who had ceased smoking for six months or more were categorized as nonsmokers.

Data were gathered, edited, coded, and added to the software SPSS Version 22.0 (IBM Corp., Armonk, NY, USA). When the quantitative data were parametric, they were shown as means, standard deviations, and ranges; when they were non-parametric, they were shown as medians and interquartile ranges (IQR). Additionally, percentages and figures were used to represent qualitative characteristics. When two independent groups had normally distributed data, the independent t-test was used; when two dependent groups had normally distributed data, the paired t-test was used; and when two independent groups had non-normally distributed data, the Mann-Whitney U test was used. These inferential analyses were carried out for quantitative variables. The chi-square test for proportional differences and Fisher's exact test for variables were used in inferential analysis for independent variables in qualitative data.

## Results

A total of 43,935 people visited the orthopedic department during the study period. Of those patients, 13,034 were males, making up 29.7% of total orthopedic ER visits, and of them, only 132 persons met our inclusion criteria for fragility hip fracture.

Results showed that the proportion of males, aged 40 years and older with fragility hip was 1.01%. In 27 of the 132 cases, there was a concomitant vertebral fracture. This indicates that 20.5% of the cases had an undetected vertebral fracture at the time of the hip fragility fracture, which was only discovered by screening lumbosacral plain X-rays as presented in Table 1.

**Table 1:** Frequency of fragility hip fractures and hidden vertebral fractures in males presented to the ER

Total orthopedic ER cases	No. = 43935	Percentage (%)
Males	13034	29.7%
Fragility hip fracture in males	132	1.01%
Hidden vertebral fractures in cases group	27	20.5%

Table 2 compares socioeconomic data and anthropometric measures of the recruited males with hip fragility fracture. The mean age for cases with hip fragility fracture was 74.32 (SD 10.8), whereas the mean age for patients with vertebral fracture was 69.59 (SD 12.96), and that for patients with no vertebral fracture was 75.53 (SD 9.83) and that was statistically significant (p=0.03). The mean weight among hip fragility fracture cases was 78.7 (SD 9.4) kg, whereas the mean weight for those with vertebral fracture was 76.7 kg (SD 8.53), and 79.22 kg (SD 9.56) for those with no vertebral fracture. The mean BMI for the study sample was 25.81 (SD 2.75), and the mean BMI for those with vertebral fractures was 25 (SD 2.9), whereas the mean BMI for those with no vertebral fractures was 26 (SD 2.7) and that was statistically significant (p=0.083).

**Table 2:** Socioeconomic data and anthropometric measures of males with hip fragility fracture. Quantitative data are expressed as mean + SD; Number in parentheses adjacent to the actual number indicates percentage of cases. Case: Patients with hidden vertebral fracture. Control: Patients with no hidden vertebral fracture. Student’s t-test for continuous variables and Pearson’s Chi-square test for categorical variables.

Variable		Hip Fragility Fracture (n = 132)	Hidden Vertebral Fracture (n= 27)	No Hidden Vertebral Fracture (n = 105)	P-value
Age	Mean (SD)	74.32 (10.77)	69.59 (12.96)	75.53 (9.83)	0.03
	Range	41-92	56-92	66-84	
Education	Non-educated	37 (28%)	6 (22.2%)	31 (29.5%)	0.877
	Low education	6 (4.5%)	1 (3.7%)	5 (4.8%)	
	Mid education	40 (30.3%)	9 (33.3%)	31 (29.5%)	
	High education	49 (37.1%)	11 (40.7%)	38 (36.2%)	
Marital status	Not married	42 (31.8%)	5 (18.5%)	37 (35.2%)	0.096
	Married	90 (68.2%)	22 (24.4%)	68 (75.5%)	
Smoking	Non-smoker	105 (79.5%)	23 (21.9%)	82 (78.1%)	0.415
	Smoker	27 (20.5%)	4 (14.8%)	23 (21.9%)	
Alcohol	No	132 (100%)	27 (100%)	105 (100%)	---
	Yes	0 (0%)	0	0	
Weight	Mean (SD)	78.7 (9.38)	76.7 (8.53)	79.22 (9.56)	0.199
	Range	63-90	68-84	70-88	
Height	Mean (SD)	174.62 (6.58)	175.26 (6.24)	174.46 (6.69)	0.651
	Range	160-185	169-181	168-180	
BMI	Mean (SD)	25.81 (2.75)	25 (2.9)	26 (2.7)	0.083
	Range	20.4-32.7	23-27	24-28	

Also, our results showed that 28% of cases with hip fragility fracture did not receive any formal education, and six (22.2%) of them had hidden vertebral fracture. Of our patients, 68.2% were married, and 24.4% of them had concomitant hidden vertebral fractures.

In this study, Table 3 demonstrates that chronic co-morbid conditions (diabetes, hypertension, ischemic heart disease, cerebrovascular stroke, congestive heart failure, atrial fibrillation, dementia, rheumatoid arthritis, chronic kidney diseases, chronic liver disease, thyroid disease, parathyroid disease, number of chronic conditions, intake of steroids, and intake of anti-epileptics) had no statistically significant differences throughout the two groupings of the study except for diabetes (p=0.054), whereas the chronic use of steroids and anti-epileptics (p=0.073), and the number of co-morbid conditions (p=0.017) showed statistically significant differences between the two groups. Results also showed that diabetes (60.6%) and hypertension (54.5%) were among the most prevalent co-morbid conditions in the study sample, followed by cerebrovascular stroke (47.7%), and chronic liver diseases (47.7%).

**Table 3:** Comparison between the two groups regarding chronic co-morbidity Quantitative data are expressed as median and interquartile range; Number in parentheses adjacent to the actual number indicates percentage of cases. Case: Patients with hidden vertebral fracture. Control: Patients with no hidden vertebral fracture. Mann-Whitney U test for continuous variables and Pearson’s Chi-square test for categorical variables.

Variable		Patients Group (n = 132)	Hidden Vertebral Facture (n = 27)	No Hidden Vertebral Fracture (n = 105)	P-value
Diabetes Mellitus		80 (60.6%)	12 (44.4%)	68 (64.8%)	0.054
Hypertension		72 (54.5%)	16 (59.3%)	56 (53.3%)	0.581
Ischemic Heart Disease		42 (31.8%)	5 (18.5%)	37 (35.2%)	0.096
Cerebrovascular stroke		63 (47.7%)	10 (37.0%)	53 (50.5%)	0.212
Congestive Heart Failure		39 (29%)	7 (25.9%)	32 (30.5%)	0.644
Atrial Fibrillation		41 (31.1%)	8 (29.6%)	33 (31.4%)	0.857
Dementia		34 (32.6%)	7 (25.9%)	36 (34.3%)	0.408
Rheumatoid Arthritis		21 (15.9%)	6 (22.2%)	15 (14.3%)	0.315
Chronic Kidney Disease		46 (34.8%)	9 (33.3%)	37 (35.2%)	0.853
Chronic Liver Disease		63 (47.7%)	14 (51.9%)	49 (46.7%)	0.630
Thyroid		32 (24.2%)	7 (25.9%)	25 (23.8%)	0.819
Parathyroid		12 (9.1%)	1 (3.7%)	11 (10.5%)	0.275
Steroids and anti-epileptic use		40 (30.3%), 18 (13.6%)	12 (26.7%), 4 (22.2%)	28 (44.4%), 14 (77.7%)	0.073
No. of chronic co-morbid conditions	Median (IQR)	4 (3 - 6)	4 (3 - 5)	5 (3 - 6)	0.017
	Range	0 – 8	2 – 7	0 – 8	

According to the multivariate regression analysis, the number of co-morbid disorders (p=0.017), hypertension (p=0.017), and chronic drugs (steroids and anti-epileptics) (p=0.009) were significant predictors of vertebral fractures among patients with fragility hip fractures as shown in Table 4.

**Table 4:** Logistic regression analysis for predictors of hidden vertebral fractures. Note: p-value > 0.05 is NS, p-value < 0.05 is significant, and p-value < 0.01 is HS.

Risk Factor	Odds Ratio	95% Confidence Interval	P-value
No. of chronic comorbid conditions	0.64	0.45 - 0.92	0.017
Hypertension	4.09	1.13 - 14.81	0.032
The chronic use of steroids and antiepileptics	1.55	1.12 - 2.15	0.009

## Discussion

El Demerdash Hospital is a tertiary health care center considered a major referral center from surrounding hospitals and other governorates in Egypt. Thus, the collected sample could be considered highly representative for the assessment of characteristics of fragility hip fractures and determining the percentage of males with fragility hip fractures who also had concurrent hidden vertebral fractures.

Concerning the prevalence of fragility hip fractures in Egypt and the main risk factors for these crippling fractures, not much information is known. Given the continuous shift towards a super-aged society, preventing hip fractures is a public health priority in our culture.

In this study, 20.5% of male patients presenting with fragility hip fractures also had a concomitant vertebral fracture. Our findings were comparable to that of Sadat-Ali et al. [15], who reported a vertebral fracture in 24.7% of the patients in Saudi Arabia. Gallacher et al. and Chinoy and Javed also found that 25% of patients presenting with non-vertebral fracture also had a simultaneous, asymptomatic vertebral fracture [16,17].

In this study the mean age for the study sample was 74.32 (SD 10.8), making age a highly significant risk factor for fragility hip fractures. This accords with other studies evidencing increasing age as a major risk factor for non-pathological fragile fractures, such as Al-Algawy et al. in 2019, who discovered that older people made up the majority of the group studied - age group of 70-79 [18].

The mean weight was 78.7 (SD 9.4) kg, and the range of BMI was 20.37 to 32.66. This is consistent with Salamat et al., who found that both BMI and weight were related to bone mineral density, and obesity significantly affects the risk for osteoporosis in men. Salamat et al. also found that the relationship between bone mineral density and weight was stronger than between BMI and bone mineral density [19].

We also found that 68.2% of our patients are married, consistent with Al-Algawy et al., whose findings showed that more than half of their patients were currently married (52%) and living with their spouses [18]. In contrast, other studies reported an association between living alone and an increased risk of fracture [20,21]. In one meta-analysis, it was estimated that those living alone had a twofold increase in fracture compared with those who were cohabiting [22]. This may be explained by different sociocultural values between different communities as typically, women are younger than their spouses or male cohabitants in Muslim religious and Arab countries, and that women also are more likely than men to age alone and to live in single-parent households. Also, the life expectancy for females is higher than that for males, making less males to be widowed.

This study found a favorable correlation between a fragility hip fracture and low educational attainment. In line with findings from comparable studies conducted overseas, about 28% of our patients had never attended any kind of formal schooling [22]. Although hip fractures are considered a serious public health concern, there hasn't been much research done on the connection between hip fractures and socioeconomic status. Some studies have connected low socioeconomic position and low levels of education to an increased risk of hip fractures [23,24], whereas other studies have found a decreased risk. We may conclude that while socioeconomic level was a key contributing factor, not all research found a correlation between hip fractures and it [22].

Numerous medical disorders are thought to increase an older adult's chance of developing a fragility hip fracture, such as hypertension and the use of anti-hypertensive drugs, diabetes, AF (atrial fibrillation), and dementia [20]. Our findings are in accord, as some of these co-morbid conditions were prevalent in our study sample [diabetes (60.6%), hypertension (54.5%), cerebrovascular stroke (47.7%), and chronic liver diseases (47.7%)]. This was confirmed again by Al-Algawy et al. [18], who discovered a strong correlation between these medical conditions and older people's elevated risk for hip fractures. Additionally, our results align with those of other investigators like Ribeiro et al. in south Brazil, and that of the Australian Institute of Health and Welfare in 2010 [25-27]. Although these co-morbid conditions were prevalent in our study sample, we could not find a statistically significant correlation regarding vertebral fractures.

In our study, we studied the pattern of participants’ lifestyles (specifically, alcohol use and smoking). Our study found that 79.5% of patients were nonsmokers. In this study, there was no significant association between smoking and fragility hip fracture. This goes against other studies that considered smoking a greater risk factor for hip fracture [28, 29]. However, our results were consistent with those of Al-Algawy et al. in Iraq, who found that 61.3% of their cases were nonsmokers [18].

Furthermore, anti-epileptic and steroid use may be involved in bone fragility among patients with rheumatoid arthritis and epilepsy. Therefore, together with other studies [30-32], the results of our study through multivariate regression analysis showed that patients using steroids and anti-epileptics are at a higher risk of vertebral fragility fractures.

The study had two limitations. First, the data were collected from a single center. However, ASUH is regarded as one of the top providers of tertiary healthcare in Egypt. Second, the study recruited only a relatively small number of participants because of COVID-19 restrictions, where only critical cases were allowed in the ER. Despite these shortcomings, the study provides a foundation for future research in this area by identifying potential risk factors of fragility fracture in Egyptian males.

## Conclusions

The study participants were divided into two groups: patients with fragility hip fractures who also had concomitant hidden vertebral fractures, and patients with fragility hip fractures who did not have any vertebral fractures. The aim of the research was to ascertain the frequency of hidden fragility vertebral fractures and the risk variables that are linked to them in patients who initially presented with fragility hip fractures. The included patients demonstrated statistical significance between the two study groups; the group with hidden vertebral fracture had more co-morbid conditions, hypertension, and chronic medication use (anti-epileptics and steroids), all of which can be considered potential risk factors for vertebral fractures. These findings emphasize the significance of determining concomitant medical history and the chronic medication use that is associated with them as potential risk factors for fragility fractures, particularly vertebral fractures.
